# The influence of gap size on the development of fracture union with a micro external fixator

**DOI:** 10.1016/j.jmbbm.2019.07.015

**Published:** 2019-11

**Authors:** Richard Meeson, Mehran Moazen, Anita Sanghani-Kerai, Liza Osagie-Clouard, Melanie Coathup, Gordon Blunn

**Affiliations:** a Division of Surgery, University College London, Stanmore, UK; b Royal Veterinary College, Hertfordshire, UK; c Mechanical Engineering, University College London, UK; d University of Central Florida, USA; e University of Portsmouth, Portsmouth, UK

**Keywords:** Interfragmentary strain, Fracture biomechanics, Rodent, Delayed-union, Non-union, Fracture healing

## Abstract

Increasingly, the rat femoral fracture model is being used for preclinical investigations of fracture healing, however, the effect of gap size and its influence on mechanobiology is not well understood. We aimed to evaluate the influence of osteotomy gap on osteotomy healing between the previously published extremes of guaranteed union (0.5 mm) and non-union (3 mm) using this model.

A femoral osteotomy in 12–14 week old female Wistar rats was stabilised with a micro fixator (titanium blocks, carbon fiber bars) with an osteotomy gap of 1.0 mm (n = 5), 1.5 mm (n = 7), 2.0 mm (n = 6). After five weeks, the left femur was retrieved. The osteotomy gap was scanned using X-ray microtomography and then histologically evaluated. The radiographic union rate (complete mineralised bone bridging across the osteotomy) was three times higher for the 1.0 mm than the 2.0 mm gap. The 1.0 mm gap had the largest callus (0.069μm^3^) and bone volume (0.035μm^3^). Callus and bone volume were approximately 50% smaller within the 2.0 mm gap.

Using cadaveric rat femurs stabilised with the external fixator, day 0 mechanical assessment of construct stiffness was calculated on materials testing machine displacement vs load output. The construct stiffness for the 1.0, 1.5 and 2.0 mm gaps was 32.6 ± 5.4, 32.5 ± 2.4, and 32.4 ± 8.3 N/mm (p = 0.779). Interfragmentary strain (IFS) was calculated using the change in osteotomy gap displacement as measured using microstrain miniature differential reluctance transducer spanning the osteotomy gap. Increasing the gap size significantly reduced the IFS (p = 0.013). The mean ‘day 0’ IFS for the 1.0, 1.5 and 2.0 mm gaps were 11.2 ± 1.3, 8.4 ± 1.5 and 6.1 ± 1.2% respectively.

A 1.5 mm gap resulted in a delayed fracture healing by 5 weeks and may represent a useful test environment for fracture healing therapy. Increasing gap size did not affect construct stiffness, but did reduce the ‘day 0’ IFS, with a doubling of non-union and halving of bone volume measured between 1.0 and 2.0 mm gaps.

## 1 Introduction

Pre-clinical experimental studies frequently use delayed or non-union models to evaluate a therapy (Garcia et al., 2013). These are typically created by either mechanical instability, damaging the vascular supply or introducing material to prevent bridging (Mills and Simpson, 2012). The most common method is to establish a critical sized defect*,* which is defined as the minimum amount of bone loss that will not heal by bone formation during the animals lifetime (Schmitz and Hollinger, 1986). Historically, studies investigating fracture biology and mechanics have been dominated by large animal models, typically sheep and goats, however the use rodent models has significantly increased to nearly 50% of all fracture studies over the last two decades (Garcia et al., 2013), and the rat is used for around one third of all in vivo fracture studies (Mills and Simpson, 2012). The size of a ‘critical sized defect’ in rats varies between studies, and reflects in part the differing mechanics of their chosen stabilisation, and whether periosteal stripping is performed. Typically, researchers have used defects of up to 8 mm and as low as 0.5 mm in rat fracture studies (Garcia et al., 2013; Mills and Simpson, 2012).

External fixators are commonly used to stabilise a defect due to their ease of application, minimal interference with subsequent analysis and their potential to alter the mechanical environment throughout the experiment. However, the literature on rodent fracture biomechanics using external fixators is limited. The most common fixators in use for rodents are the thermoplastic polyether ether ketone (PEEK) Glatt fixator from the AO Research Institute Davos (Glatt and Matthys, 2014), which is commercially available and the titanium alloy ‘Harrison style’ fixator (Harrison et al., 2003; Ho et al., 2014; Lee et al., 2005; Smitham et al., 2014). The more rigid Harrison fixator (Osagie-Clouard et al., 2019), is a unilateral uniplanar fixator with a double carbon fiber connecting bar, which has the novel function of permitting variable gap size, by sliding the adjustable distal titanium block along the bar. This approach to varying gap size maintains the pin to osteotomy gap distance irrespective of gap size, whereas other micro fixators require an ostectomy of the desired gap distance to vary said gap. Increasing osteotomy size may also influence bone healing by a potential variation in bone biology along its length (diaphyseal to metaphyseal). The Harrison style fixator has previously showed consistent union with a 0.5 mm gap and consistent non-union with a 3 mm gap with a rat femoral osteotomy after 5 weeks (Harrison et al., 2003) and in female adult wistar rats (Lee et al., 2005; Smitham et al., 2014). The AO fixator is considerable less stiff (Osagie-Clouard et al., 2019) and although studies generally use controls, direct comparison of results on the biology of fracture fixation using different fixators is probably inappropriate due to the difference in their mechanics and hence differences in healing.

Numerous studies have tested their hypotheses using osteotomy gaps in the range of 1–2 mm in rats, however the biomechanics have only been evaluated with FE modeling (Wehner et al., 2014). Currently, no studies have made a sequential evaluation of intermediary gap sizes between guaranteed healing, delayed union and non-union, to identify the point at which delayed union occurs. Inherently, the biomechanics of the fixator, including the fracture (osteotomy) gap interfragmentary strain (IFS) (Perren, 1979), and overall construct stiffness, will affect the outcome. In order to understand the findings from one study to another, evaluation of the fracture biomechanics would be highly informative.

Clinical fractures that heal more slowly than expected are termed delayed unions and some may fail to heal at all and are termed non-unions. Many pre-clinical studies evaluate interventions in models that go on to successful union, and therefore may not be an appropriate test scenario. Likewise, the non-union pre-clinical model may be too challenging to demonstrate efficacy of a new treatment and therefore the delayed union may a useful test environment in pre-clinical studies.

The hypothesis for our study was that a delayed union type healing would be seen in a gap size midway between the published established union at 0.5 mm and non-union at 3 mm when using the Harrison style fixator at 5 weeks (Harrison et al., 2003; Ho et al., 2014; Smitham et al., 2014). The objectives were to assess the fracture healing with three intervening gap sizes and to determine the potential variation in initial mechanical environments in terms of construct stiffness and interfragmentary strain.

## 2 Methods

### 2.1 Fixator design & application

**Fig. 1:**
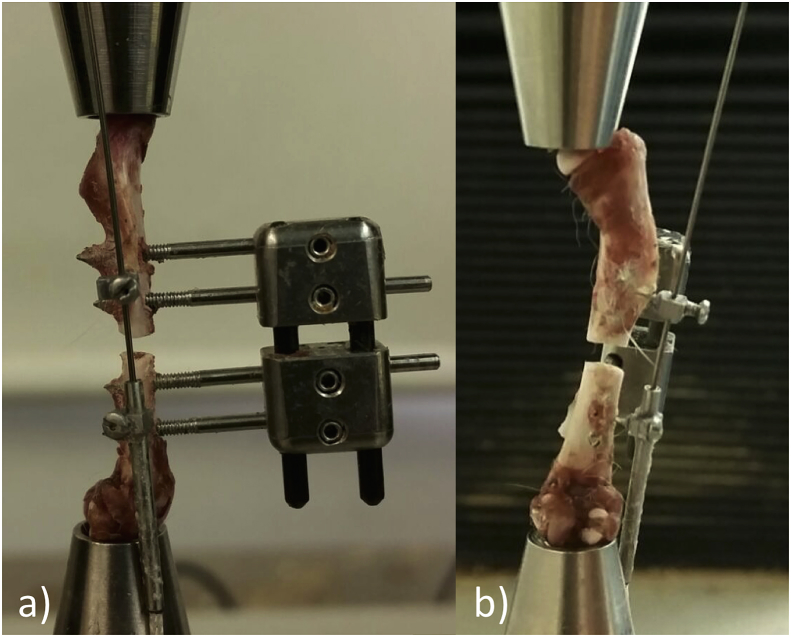
Ex-vivo femur loaded from femoral head to condyles in a materials testing machine with a cranially applied Harrison style fixator. A Lord microdisplacement sensor was applied to the lateral surface (1a = lateral view, 1b = caudal view).

The Harrison style fixator is a unilateral uniplanar (Type Ia) external fixator with two transcutaneous intraosseus pins proximal and two pins distal to a surgically created osteotomy. It has a double connecting bar (2 mm diameter carbon-fiber; epoxy resin matrix bars) with two titanium connecting blocks which can slide axially along the bar, and secured in position using miniature grub screws, allowing alteration of the osteotomy gap size (Fig. 1). This gives a consistent positioning of the pins in the bone and a consistent distance from the osteotomy, but varies the bar working length (bar length between the two fixator blocks), as the osteotomy is increased.

Female Wistar rats, 12–14 weeks old (230–300 g) had the fixator placed on the left craniolateral femur following a lateral surgical approach (Harrison et al., 2003). Using a precision jig-guide, four bicortical 1.4 mm diameter end-threaded self-tapping stainless steel pins were placed in predrilled 1.0 mm holes in a cranial to caudal orientation. Consistent proximodistal positioning was based on the distal extent of the greater trochanter. Pins were exited through separate skin incisions and the custom variable spacing fixator was attached, using a precision spacer to ensure a fixed distance between the near cortex and connecting blocks of 9 mm. A mid-diaphyseal femoral osteotomy, with no periosteal stripping was made using a diamond tipped hand-saw, whilst applying sterile saline coolant/lubricant. Rats were then randomly assigned to have a 1.0 mm, 1.5 mm or 2.0 mm osteotomy gap using an appropriately sized precision spacer placed between the ends of the osteotomised bone, and the grub screws were tightened. The biceps femoris was closed over the osteotomy with a single horizontal mattress suture (1.5M PDS II, Ethicon), and the skin was closed with an intradermal continuous suture (1.5M monocryl, Ethicon). Analgesia was provided with subcutaneous administration of buprenorphine 0.05 mg/kg prior to surgery, then three times daily for 48 h per os, within a sweetened jelly. Activity was unrestricted post surgery for 5 weeks until euthanasia. All procedures were carried out in accordance with the Animals Scientific Procedures Act 1986, were approved by the University's Animal Welfare Ethical Review Board and were aligned to the ARRIVE guidelines. Those taking part in any surgical procedure held UK Home Office licences.

### 2.2 X-ray microtomography (MicroCT) and radiography

**Fig. 2:**
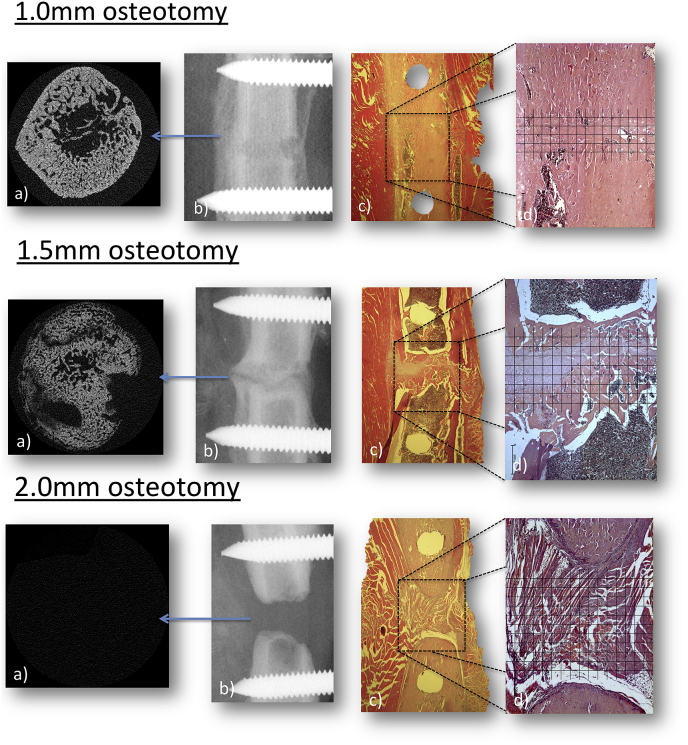
Representative images from the analysis of healing for each fracture gap size. a) Shows the central transverse 5 μm thick slice from the centre of the osteotomy from microCT analysis. b) Shows a lateral-medial radiograph centred over the two innermost fixator pins and the osteotomy. c) Shows a 1x magnification image of the central sagittal slice, Hematoxylin and Eosin stained. d) Shows a 2.5x magnification image of the central region of the femur with the histomorphometric grid applied for quantitative morphometry.

After 5 weeks, the left femur with the fixator in place was retrieved. In order to reduce microCT beam-hardening artifact generated from the interaction of the X-ray beam and the metallic implant, a radiolucent PEEK fixator block was connected externally to the fixator pins after careful removal of the skin with surrounding soft-tissues, and then without disturbing the fracture callus the titanium block fixator was then removed. Samples were fixed in 10% buffered formaldehyde for up to three days. The formalin fixed samples were wrapped in cling-film to prevent dehydration and mounted into a sample holder for microCT scanning. Samples were scanned using a Bruker Skyscan 1172 micro-tomograph (Bruker, Belgium), at 60 KV, 167μA with a 0.5 mm aluminum filter. A rotation step of 0.5°, without frame averaging, and an image pixel size of 4.89μm was used. A single image capture image was taken with the image intensification ‘scout’ prior to scanning, for 2D radiographic assessment of the osteotomy union. Radiographic scouts were randomised and blinded to score healing according to the AO-ASIF recommendations for long bone fractures; united, not united or uncertain (Müller et al., 1979) as follows: *Ununited* (Fig. 2, 2.0 mm osteotomy b)) where there was no mineralised tissue bridging between the ends of the osteotomy; *uncertain* (Figs. 2 and 1.5 mm osteotomy b)) where there was new bone formation, however a radiolucent line remained between the proximal and distal segments, and *united* (Figs. 2 and 1.0 mm osteotomy b)) where no gap between bone ends was visible.

MicroCT scans were reconstructed using NRecon (Bruker, Belgium) with smoothing = 2, ring artifact reduction = 12% and beam hardening artifact = 41%. Analysis was performed with CTAn (Bruker, Belgium). Using the measuring tool, the centre point of the osteotomy was determined and the transverse slice at that point was selected as the reference slice. The callus was isolated using a 2D ROI shrink wrap stretching over holes <40 pixels, despeckled <150 voxels and then 3D analysis was performed. In order to make a direct comparison of healing between the differing gap sizes, the central 60% of the osteotomy gap. i.e. only new bone formation within the osteotomy was analysed for each size, which translated to 120, 180 and 240 slices at 5 μm slice thickness, giving 0.6 mm, 0.9 mm and 1.2 mm osteotomy gap analysis for the 1.0, 1.5 and 2.0 mm gap respectively. Where absolute measures were made in quantitative morphometrics, such as total bone volume (BV), these were divided by the number of slices contributing the analysis for each gap size, to allow for a direct comparison of bone formation despite analysing different volumes.

### 2.3 Histology

Following CT imaging, bones were decalcified in a 12.5% solution of ethylenediaminetetraacetic acid then sequentially dehydrated for 24 h, followed by de-fatting with chloroform for 48 h and embedded into wax, with the fixator pins orthogonal to the facing surface of the block. Fixator blocks and pins were removed once the wax had set and a microtome (ThermoFisher Scientific, UK) was used to make 5 μm thick slices. The alignment of the blocks within the microtome was altered as necessary to ensure a central sagittal slice through the femur. The position of a mid-sagittal section through the fracture gap was assessed using the fixator pin tract holes. Wax slices were mounted onto positively charged glass slides (X-tra, Leica biosytems, UK), de-waxed and then hydrated. Samples were then stained with Hematoxylin (Sigma-Aldrich, UK) nuclear stain for 5 min. Excess stain was removed by gentle washing with water for 5 min. Slides were counterstained in 1% Eosin (Sigma-Aldrich, UK) for 4 min and then washed and dehydrated in increasing concentrations of alcohol. Slides were cleaned in xylene and mounted under 40 mm coverslips using Pertex Mounting Medium (CellPath plc, UK).

### 2.4 Histomorphometric analysis

Slides were observed under a light microscope (KS-300 Zeiss, UK). Histomorphometric analysis at 2.5x magnification was performed on the most central slice, using a line-intercept method with a grid scaled to the graticule and drawn using PowerPoint (Microsoft, USA). The grid covered the entire visual field from top to bottom (lateral to medial cortex) and was centred over the osteotomy; its width was equivalent to the original 1.0, 1.5 or 2.0 mm osteotomy. Grid squares were 160 μm in both directions and intersections, giving 75, 120 and 165 intersections evaluated for the 1 mm, 1.5 mm and 2.0 mm gaps respectively. Intersections were then scored as bone, cartilage, fibrous tissue, vascular (red blood cells seen not within tissue matrix) or void based upon Hematoxylin and Eosin uptake and cell morphology to provide a percentage tissue formation.

### 2.5 Assessment of fixator biomechanics and immediate IFS at day 0

The fixator was placed as per the surgical description on the femora of cadaveric 18–20 week old Wistar rats (n = 4). Femora with the fixator still attached were then disarticulated at the hip and stifle and stripped of soft-tissue attachments. An orthogonal (lateral to medial orientated) 0.8 mm bicortical hole was drilled between the two proximal and two distal fixator pins. A microminiature differential variable reluctance transducer (DVRT - accuracy 0.001 mm) (Lord MicroStrain, model 6101-0200, Williston, USA) was then inserted and fixed in position using cyanoacrylate glue, to quantify fracture movement (Fig. 1). Femurs were biomechanically tested using a materials testing machine (Zwick Roell 5T, UK). They were mounted in an axial loading jig with the femoral condyles centred over the lower mount and the upper mount was centred over the femoral head to simulate a physiological loading axis of the femur along its mechanical axis. This set-up effectively tested the entire construct of fixator and bone as a single unit. Three gap sizes were evaluated per specimen; 1.0 mm, 1.5 mm and 2.0 mm. The distal fixator connecting block was loosened to allow insertion of the precision titanium spacer and then tightened again. The space was then checked a second time prior to loading and again between each repeat by ‘offering-up’ the spacer to the gap. Care was taken to ensure the gap was even across the width of the osteotomy.

The peak vertical force for each hind limb in rats is 60% bodyweight at the walk (Clarke, 1995). A maximum weight of 300 g for an individual rat was seen in the in vivo study and therefore peak-walking load was assumed to be 1.8N. A single cycle non-destructive test was performed, with a preconditioning load of 0.5N, followed with loading to a maximum of 10N in compression at 5 mm/min, sampling rate of 50 Hz. The first cycle was disregarded and then four repeats were performed per gap size, per sample. The sensor (DVRT) output (i.e. millivoltage changes) was recorded and the difference pre and at peak load was determined. This was then converted into a displacement according to manufacturers calibration equation. The pre load and peak load lengths were then used to calculate IFS based on change in length divided by the original length. Fixator–bone construct stiffness was determined from the load-displacement graphs obtained from TestXpert software (Zwick, Roell, UK). A linear regression line (r^2^) was calculated for the linear portion and r^2^ > 0.99 was considered appropriate for the linear elastic region. The gradient (m) was determined based on a y = mx + c equation and gave the stiffness.

### 2.6 Statistical analysis

Fishers Exact was used to compare the fracture healing outcome. Normality was determined using a Shapiro Wilk test and non-parametric tests were performed to compare groups using Kruskal-Wallis (KW), and Mann-Whitney U (MWU) performed with Bonferroni correction applied (alpha = 0.05/number of comparisons). Results were expressed as means ± standard deviations. Tests were analysed with SPSS version 24 (IBM, Chicago, USA).

## 3 Results

### 3.1 Radiographic and microCT assessment of healing

**Fig. 3:**
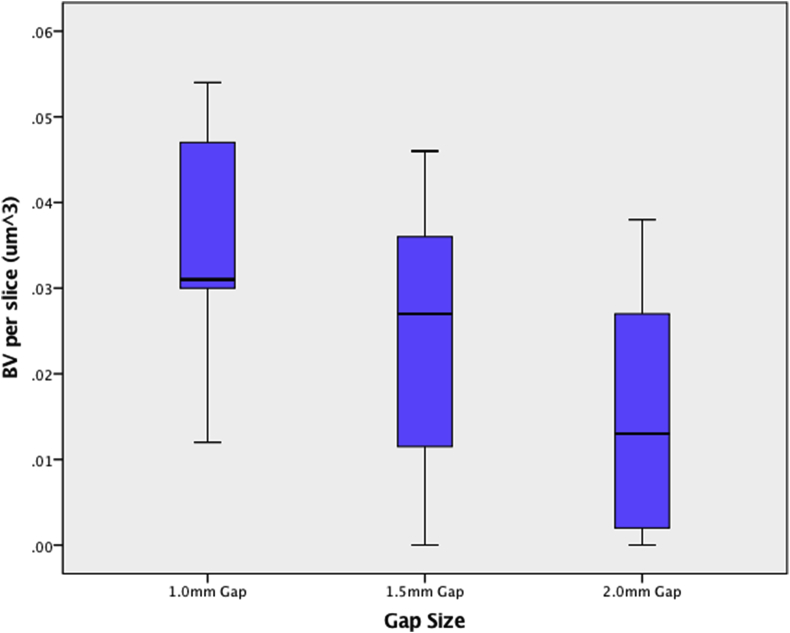
Boxplot showing (the average per 5 μm slice) microCT bone volume (BV um^3), with the BV reducing sequentially as the gap size increases.

As the gap size increased there was an increase in the AO classification of ununited and uncertain fracture classifications and a concomitant decrease in united rates, with the ununited rate more than doubling (Table 1, Fig. 2b), however this was not significantly different with Fishers Exact comparison. On MicroCT quantitative morphometric analysis, the 1.0 mm gap size had a larger callus volume (0.069 ± 0.04μm^3^) and bone volume per slice (0.035 ± 0.02μm^3^); than for the 2.0 mm gap size (0.029 ± 0.03 and 0.026 ± 0.02μm^3^ respectively - Fig. 2, Fig. 3). Tissue surface area per slice, giving an index of callus size, was higher in the smallest 1.0 mm gap (0.41 ± 0.22μm^2^) than the largest 2.0 mm (0.14 ± 0.12μm^2^). The measured trabecular thickness was higher in the smaller 1.0 gap than the larger 1.5 mm gap (0.055 ± 0.01μm and 0.044 ± 0.01μm), however it increased again when the gap size increased to 2.0 mm (0.057 ± 0.02μm). Full microCT results are summarised in Table 2.

**Table 1:** Global radiographic scoring of fracture healing at 5 weeks based on the A0-ASIF system.

Gap Size (mm)	Ununited	Uncertain	United
1.0	1/5 (20%)	1/5 (20%)	3/5 (60%)
1.5	3/7 (43%)	2/7 (29%)	2/7 (29%)
2.0	3/6 (50%)	2/6 (33%)	1/6 (17%)

**Table 2:** MicroCT quantitative morphometry indices of bone formation within the 60% of the osteotomy gap where TV (um^3) = tissue volume, BV (um^3) = bone volume, TV/BV (%) = percentage bone volume, TS (um^2) = tissue surface, BS (um^2) = bone surface, Tb.Th (um) = trabecular thickness, Tb.Sp (um) = trabecular separation, Tb.N (1/μm) = trabecular number.

	1.0 mm Gap	1.5 mm Gap	2.0 mm Gap
TV per slice (um^3)	0.07 ± 0.04	0.05 ± 0.03	0.03 ± 0.03
BV per slice (um^3)	0.04 ± 0.02	0.02 ± 0.02	0.02 ± 0.02
BV/TV (%)	54.25 ± 9.38	53.79 ± 20.82	66.39 ± 15.37
TS per slice (um^2)	0.41 ± 0.23	0.35 ± 0.25	0.14 ± 0.12
BS per slice (um^2)	2.09 ± 1.62	1.81 ± 1.22	0.90 ± 0.94
Tb.Th (um)	0.06 ± 0.01	0.04 ± 0.01	0.06 ± 0.02
Tb.Sp (um)	0.12 ± 0.05	0.07 ± 0.03	0.07 ± 0.05

### 3.2 Histomorphometric analysis

**Fig. 4:**
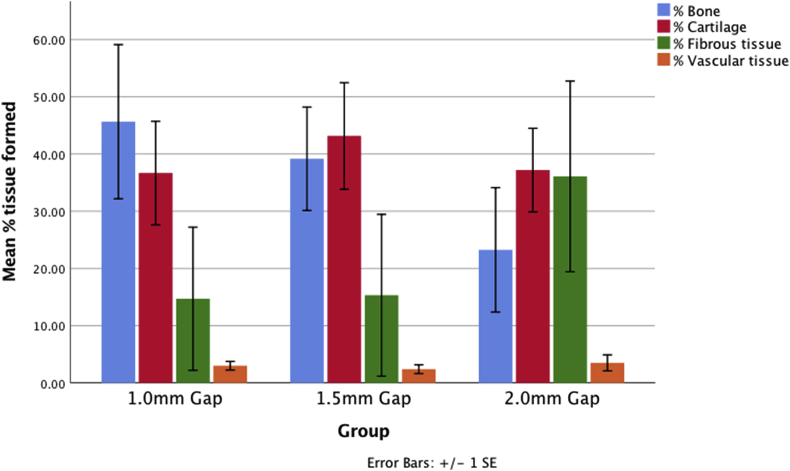
Quantitative morphometric data from the central region of the osteotomy, from the 2.5x magnification Hematoxylin and Eosin stained slides, showing the mean ± SEM reduction in % bone formation as the gap size increases, with the 1.5 mm gap showing a concomitant increase in cartilage tissue, but the 2.0 mm showing a concomitant increase in fibrous tissue.

As gap size increased, the area occupied by bone within the callus decreased, and fibrous tissue increased (Fig. 2c and d). Cartilage tissue was highest in the mid-sized gap, however, the amount of fibrous tissue was still lower than the biggest gaps. None of these trends were statistically significant (Table 3 and Fig. 4), however clear trends were identified.

**Table 3:** Quantification of tissue formed within the gap as percentage total tissue from line intercept analysis of Hematoxylin and Eosin stained mid sagittal sections.

% TISSUE	1.0 mm Gap	1.5 mm Gap	2.0 mm Gap
Bone	45.6 ± 33.0	39.1 ± 23.9	23.2 ± 26.6
Cartilage	36.7 ± 22.1	43.1 ± 24.6	37.2 ± 17.9
Fibrous	14.7 ± 30.6	15.3 ± 37.4	36.1 ± 40.8
Vascular	3.0 ± 1.9	2.4 ± 2.0	3.5 ± 3.4

### 3.3 Mechanical analysis

**Fig. 5:**
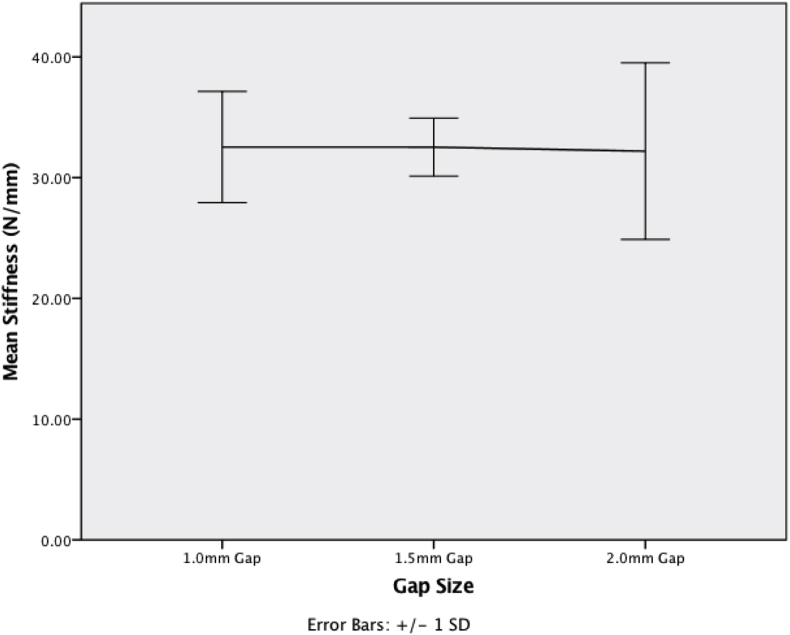
Line graph showing the mean ± SD construct stiffness (N/mm) measured, with no significant change as the gap size increased.

**Fig. 6:**
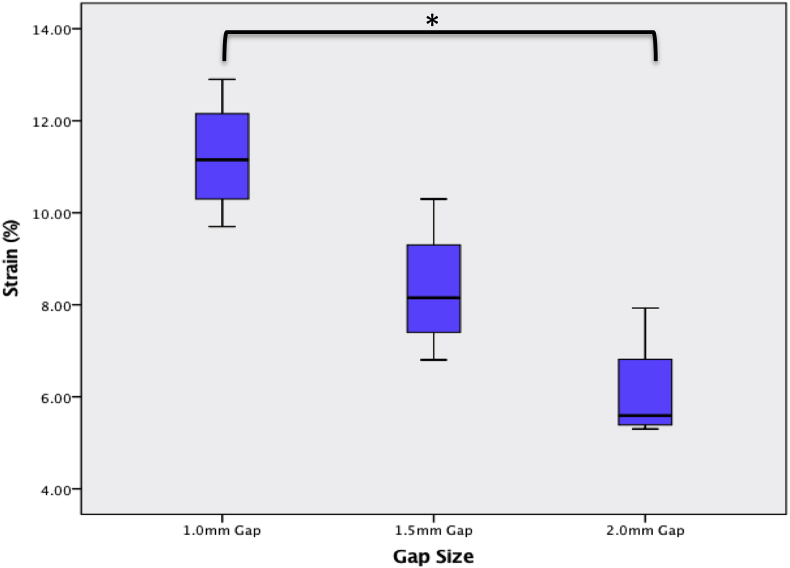
Boxplot showing the change in day 0 immediate IFS (%) as the gap size increased.

The mean ± SD stiffness of the four osteotomised femurs with the fixator in situ for the 1.0, 1.5 and 2.0 mm gaps were 32.6 ± 5.4, 32.5 ± 2.4, and 32.4 ± 8.3 N/mm (Fig. 5); the gap size over the ranges tested had no impact on the construct stiffness (p = 0.779), however gap size did significantly reduce the IFS in the gap (p = 0.013), (Fig. 6). The mean ± SD % IFS for the 1.0, 1.5 and 2.0 mm gaps were 11.2 ± 1.3, 8.4 ± 1.5 and 6.1 ± 1.2% respectively.

## 4 Discussion

Using the rigid Harrison style micro external fixator, this study demonstrated a predominant delayed union scenario with an osteotomy gap of 1.5 mm after 5 weeks, when compared with previously published studies using the same fixator and a 0.5 mm gap (Harrison et al., 2003; Smitham et al., 2014). This study also showed a 1 mm gap leading to a predominance of union and the 2 mm resulting in a delayed union with an atrophic appearance, indicating non-union, but our study duration was not of sufficient length for an unequivocal definition. Within each group there was greater variation in healing pattern than shown in the published 0.5 mm and 3 mm gaps. Most of 2 mm fracture gaps had an atrophic style non-union with medullary capping and a fibrous tissue connection, however it must be considered that a longer duration study would be required to fulfill current time definitions of delayed union (Garcia et al., 2013). This study had an end point of 5 weeks to allow comparison to previous studies that used the same fixator and showed a non-union with a 3 mm and union with a 0.5 mm osteotomy and the same fixator (Harrison et al., 2003; Ho et al., 2014; Smitham et al., 2014). Under normal circumstances, rat femoral fracture healing should be achieved by 5 weeks, therefore lack of union indicates delayed or non-union at this stage. Uncertain and un-united radiographic categories are determined by the radiographic appearance of the fracture are technically both delayed union, as our study is not of sufficient duration to use the term non-union, and hence it was avoided. A longer study with sequential culling may have given more information on the rate of healing. This would have allowed us to understand whether fracture healing is reduced by increasing the gap or totally arrested, however in terms of being informative for rodent fracture healing studies with typically end points of 5–6 weeks, this was considered unnecessary, and would have used more animals, contrary to the principles of the 3Rs.

The fixator used in this study has been shown to be significantly stiffer at 4.7 times the axial stiffness of the commercially available AO fixator (Osagie-Clouard et al., 2019), and hence will have provided a relatively more rigid fixation. Interestingly, increasing the fracture gap, which increases the working length of the carbon fiber bars did not have any statistically significant effect on construct stiffness, indicative of the relatively rigid fixator design compared with the physiological forces it withstands. Very minor influence on stiffness is possible, however the group sizes required to determine if extremely small changes were statistically significant would be prohibitively large. This is useful as it provides an ability to investigate the influence of gap size in terms of its biological impact and the variation in IFS, without influencing construct stiffness.

The impact of gap size on the healing in this particular model system may be driven by the biological impact of the gap size on tissue healing, rather than its mechanical effects. Large animal models have shown that increased fracture gaps with the same IFS had reduced vascularisation and hence diminished biological ability to heal (Claes et al., 2003). However, other studies quantifying blood vessel formation have shown no difference between atrophic non-unions, hypertrophic non-unions and healing fractures (Reed et al., 2002), although vessels appear at a later stage and therefore early vascularisation may be key (Reed et al., 2003). The histology in this study also showed a consistent level of vascularisation between different gap sizes and their subsequent healing fates. However, the histologic analysis was performed at five weeks and therefore it is conceivable with an increasing gap size that the time required for vascular development could be longer and perhaps critical blood vessel density it not reached at a sufficiently early time frame.

Despite the commonplace role of rodents in fracture healing research, most studies have evaluated the influence of IFS on fracture healing with large animal models in vivo (Claes et al., 2003, 1997; Claes and Heigele, 1999) or using FE model (Comiskey et al., 2010; Steiner et al., 2014; Wehner et al., 2014). With the increasing use of rodents in bone healing studies, an understanding of the mechanical environment is needed in rodents. This is the first time such measures have been directly and accurately measured in an ex vivo study in rats, with a micro-miniature differential variable reluctance transducer (accuracy 0.001 mm). The use of a highly sensitive displacement transducer should give a more accurate measure than those based on the materials testing machine actuator displacement. However, we acknowledge that the transducer is measuring displacement in the axis of the transducer and this could vary across the bone gap itself. Additionally, the exact femoral alignment would also differ in vivo, but approximations are required to test in a material testing machine. The in vitro tests to measure IFS were carried out with the load axially aligned. Due to the orientation of the femur in the live animal, bending and torsional moments would induce strain. Alignment of the transducer along a different plane on the femur again may have produced differing results, however our tests showed that a reduction in IFS was related to an increase in delayed union indicating that the IFS may be an oversimplification. Critically, the set-up considerations noted are consistent across the gaps tested, and hence their comparison is still informative. Future studies could make consideration of multiple gauge assessment to build a composite assessment of interfragmentary motion. It would also be useful to make an ex and in vivo comparison this fixator to AO/Glutt fixator for healing over different gap sizes.

It should also be noted that the cadaveric femurs were in the 18–20 week range whereas the in vivo study rats were 12–14 weeks old. This was in part due to a consideration of 3Rs, and although the physes remain open throughout these ages (Roach et al., 2003), growth is substantially decelerating, and overall limb length was not expected to change much. Furthermore, the IFS was calculated using a displacement gauge and fixator which was placed at a standard distance from the osteotomy irrespective of the overall femoral length, hence creating a consistent biomechanical environment.

In a system where the fixator stiffness is unaffected by increasing gap size, and hence the change in gap length for a given load is consistent, IFS will arithmetically reduce as the denominator gap size increases. However, assessment of the initial IFS did not indicate the subsequent pattern of healing as predicted by Perren's IFS theory of fracture healing (Perren, 1979). IFS theory predicts for a given interfragmentary movement, the bigger the gap, the lower the IFS, if all other factors remain unchanged. However, large gaps and critical sized defects, even when fixed very rigidly do not heal, and consistent with these findings, there was a doubling of ununited fractures and halving of bone volume, with an associated increase in cartilage in the 1.5 mm gap and fibrous tissue within the 2.0 mm osteotomy as the gap increased from 1 mm. This corresponded to a ‘day 0 equivalent’ measure of IFS from 12% to 6% respectively. Overall, the groups with the small gaps and an initial IFS >10% had improved healing than those with big gaps and an IFS <10%, suggesting gap size biological factors may overwhelm mechanical factors. Some large animal studies with known gap sizes and interfragmentary movements have also shown good bone healing with IFS >2–10% (Claes et al., 1995; Kenwright and Goodship, 1989). Claes et al. showed that a high initial IFS, above the Perren 10% threshold resulted in increased callus formation, however, a larger gap had less bone formation for the same initial strain (Claes et al., 1997). However, although initial IFS is important in the extreme, when a fracture occurs, an established sequence of events follows (Elliott et al., 2016), with an initial deposition of strain tolerate tissue, such as granulation tissue, followed by sequential deposition of more strain intolerant tissues. The wide tissue cuff or ‘callus’, seen in indirect fracture healing, stiffens the gap, and further increasing fracture stability and reducing IFS (Perren, 2015). When looking at the bone surface measures (BS) and tissue surface (TS) measures on microCT, there was a trend for a smaller callus as the strain reduced, potentially consistent with a bigger callus cuff being required when there is a higher IFS. Various models have expanded upon the work of Perren. Carter and Blenman, suggested that it is not only the amount of strain, but the way the strain is applied, be it in compression, tension, shear, and further that the degree of vascularisation plays influence (Carter et al., 1988). Their finite element model also accounted for eccentric callus formation with an asymmetric cartilage deposition, which was noted in some of the samples in this study. They suggested this was due to varying hydrostatic forces with a more ‘compressive microenvironment’ producing more cartilage and a ‘tensile’ environment would have less callus with a more fibrous character. This is consistent with the types of loading patterns that will be developed within an osteotomy of the rat femur with its eccentric mechanical axis and the use of a unilateral external fixator. Prendergast suggested a further iterative model with two biophysical stimuli; fluid velocity and shear strain components, playing a role in the solid and liquid phases (Prendergast et al., 1997). However, these are all models and typically approximate in vivo findings in their extremes.

Other complicating factors such as increasing animal age (Strube et al., 2008) or sex appear to influence fracture healing in some studies, although in a study by Mehta et al. (2010) the large difference in bodyweight between female and male rats was not controlled (Mehta et al., 2010). This study however, had a tightly controlled age range and hence weight, and all were female Wistar rats.

In conclusion, the fixator design evaluated here provides stable construct/fracture stiffness over a range of fracture gap sizes. Increasing gap size did not affect construct stiffness, but did reduce the ‘day 0’ IFS from 12 to 6%, with a doubling of the incidence of non-union and halving of bone volume measured. This is in contrast to the expected outcome based on IFS theory, but may be due to the biological impact of the gap size over and above the mechanics in this model system. This is the first study to evaluate and directly compare a range of gap sizes between guaranteed union and non-union in a rodent femoral fracture model using the Harrison style fixator, and the 1.5 mm osteotomy gap provided a delayed-union at 5 weeks. This study provides informative that will be informative to researches using Harrison style fixators for fracture healing studies in rats, and may allow for more precise selection of gap size for their investigations than the two extremes previously published (Harrison et al., 2003; Ho et al., 2014; Smitham et al., 2014).
